# Lysosomal acid lipase regulates VLDL synthesis and insulin sensitivity in mice

**DOI:** 10.1007/s00125-016-3968-6

**Published:** 2016-05-06

**Authors:** Branislav Radović, Nemanja Vujić, Christina Leopold, Stefanie Schlager, Madeleine Goeritzer, Jay V. Patankar, Melanie Korbelius, Dagmar Kolb, Julia Reindl, Martin Wegscheider, Tamara Tomin, Ruth Birner-Gruenberger, Matthias Schittmayer, Lukas Groschner, Christoph Magnes, Clemens Diwoky, Saša Frank, Ernst Steyrer, Hong Du, Wolfgang F. Graier, Tobias Madl, Dagmar Kratky

**Affiliations:** Institute of Molecular Biology and Biochemistry, Center of Molecular Medicine, Medical University of Graz, Harrachgasse 21, 8010 Graz, Austria; Center for Molecular Medicine and Therapeutics, Department of Medical Genetics, University of British Columbia, Vancouver, BC Canada; Center for Medical Research/Institute of Cell Biology, Histology and Embryology, Medical University of Graz, Graz, Austria; Institute of Pathology, Medical University of Graz, Graz, Austria; Omics Center Graz, BioTechMed-Graz, Graz, Austria; Center for Neural Circuits and Behaviour, University of Oxford, Oxford, UK; Health, Bioanalytik und Metabolomics, Joanneum Research, Graz, Austria; Institute of Biomedical Engineering, Graz University of Technology, Graz, Austria; Institute of Molecular Biosciences, University of Graz, Graz, Austria; Department of Pathology and Laboratory Medicine, Indiana University School of Medicine, Indianapolis, IN USA; Department of Chemistry, Technical University, Munich, Germany; Institute of Structural Biology, Helmholtz Zentrum, Munich, Germany

**Keywords:** Glucose tolerance, Lipolysis, Lysosomes, VLDL

## Abstract

**Aims/hypothesis:**

Lysosomal acid lipase (LAL) hydrolyses cholesteryl esters and triacylglycerols (TG) within lysosomes to mobilise NEFA and cholesterol. Since LAL-deficient *(Lal*^*-/-*^*)* mice suffer from progressive loss of adipose tissue and severe accumulation of lipids in hepatic lysosomes, we hypothesised that LAL deficiency triggers alternative energy pathway(s).

**Methods:**

We studied metabolic adaptations in *Lal*^*-/-*^ mice.

**Results:**

Despite loss of adipose tissue, *Lal*^*-/-*^ mice show enhanced glucose clearance during insulin and glucose tolerance tests and have increased uptake of [^3^H]2-deoxy-D-glucose into skeletal muscle compared with wild-type mice. In agreement, fasted *Lal*^*-/-*^ mice exhibit reduced glucose and glycogen levels in skeletal muscle. We observed 84% decreased plasma leptin levels and significantly reduced hepatic ATP, glucose, glycogen and glutamine concentrations in fed *Lal*^*-/-*^ mice. Markedly reduced hepatic acyl-CoA concentrations decrease the expression of peroxisome proliferator-activated receptor α (PPARα) target genes. However, treatment of *Lal*^*-/-*^ mice with the PPARα agonist fenofibrate further decreased plasma TG (and hepatic glucose and glycogen) concentrations in *Lal*^*-/-*^ mice. Depletion of hepatic nuclear factor 4α and forkhead box protein a2 in fasted *Lal*^*-/-*^ mice might be responsible for reduced expression of microsomal TG transfer protein, defective VLDL synthesis and drastically reduced plasma TG levels.

**Conclusions/interpretation:**

Our findings indicate that neither activation nor inactivation of PPARα per se but rather the availability of hepatic acyl-CoA concentrations regulates VLDL synthesis and subsequent metabolic adaptations in *Lal*^*-/-*^ mice. We conclude that decreased plasma VLDL production enhances glucose uptake into skeletal muscle to compensate for the lack of energy supply.

**Electronic supplementary material:**

The online version of this article (doi:10.1007/s00125-016-3968-6) contains peer-reviewed but unedited supplementary material, which is available to authorised users.

## Introduction

Lysosomal acid lipase (LAL) hydrolyses cholesteryl esters (CE) and triacylglycerols (TG), delivered to the lysosome mainly via LDL particle uptake [1], to release mono- and diacylglycerols and mobilise cholesterol and NEFA for membrane assembly, steroidogenesis and energy production. Genetic mutations of human *LAL* (also known as *LIPA*) cause an autosomal recessive lysosomal storage disorder with accumulation of CE predominantly in hepatocytes, adrenal glands, intestine and cells of the monocyte-macrophage system throughout the body [2]. Mutations in *LAL* cause Wolman disease (WD) or cholesteryl ester storage disease (CESD) [3–5]. WD is a rare, neonatal-onset disease with less than 1% of LAL activity, characterised by massive hepatosplenomegaly, adrenal calcifications, malabsorption, growth retardation and cachexia. Affected patients die within the first three to 12 months of life [2, 6, 7]. CESD patients have up to 5% residual LAL activity [8], which keeps the syndrome mostly unrecognised until adulthood. These patients suffer from progressive lysosomal CE and TG accumulations, which lead to the characteristic liver pathology and elevated concentrations of serum transaminases, serum LDL and TG. Premature death of individuals with CESD is due to liver failure and/or accelerated atherosclerosis rather than to chronic hyperlipidaemia [2, 9, 10].

In contrast to humans, complete loss of LAL activity in mice phenotypically resembles CESD rather than WD. LAL-deficient *(Lal*^*-/-*^*)* mice appear normal at birth and reach the median life span of approximately 1 year [11]. Severe accumulations of CE and TG are found predominantly in the liver, spleen, small intestine and adrenals [11–13]. *Lal*^*-*/-^ mice exhibit reduced size and body weight (BW) compared with wild-type (WT) littermates as well as progressive loss of white adipose tissue (WAT) and brown adipose tissue [12, 13]. In this study, we explored the metabolic changes caused by impaired lysosomal TG and CE hydrolysis in *Lal*^*-*/-^ mice.

## Methods

### Animals

Age- and sex-matched *Lal*^*-/-*^ and WT littermates [13] on a C57BL/6J background were maintained with unlimited access to chow and water in regular 12 h light/12 h dark cycles. For fenofibrate treatment, 5-week-old *Lal*^*-/-*^ mice were administered 0.2% fenofibrate/chow for 4 weeks. Experimenters were blind to group assignment and outcome assessment. Animal experiments were approved by the Federal Ministry of Science, Research and Economy, Vienna, Austria. See electronic supplementary material (ESM) Methods for further details.

### Lipid and hormone concentrations and fast protein liquid chromatography

Cholesterol, TG, glycerol and markers of liver injury were quantified enzymatically. Lipoprotein profiles were analysed by fast protein liquid chromatography (FPLC) [14]. Hormones were determined by ELISA. See ESM Methods for further details.

### ATP analysis

Polar metabolites from mouse liver were extracted as described previously [15]. HPLC was performed on a 1100 Agilent capillary LC (Agilent Technologies, Santa Clara, CA, USA) equipped with a polyhydroxyethyl column (PolyLC Inc, Columbia, MD, USA). Solvent A (10 mmol/l ammonium acetate/water) and solvent B (10 mmol/l ammonium acetate/90% acetonitrile) were used in varying gradients. Selected ions/fragments were detected by TSQ Quantum Ultra Mass Spectrometry (Thermo Fisher Scientific, Waltham, MA, USA) in negative mode. See ESM Methods for further details.

### De novo lipid synthesis

Animals were i.p. injected with [^14^C]acetate (5 μCi in 200 μl PBS), killed 1 h post-injection, and livers were isolated and lyophilised for 48 h. Lipid extracts were separated by thin-layer chromatography (TLC) (n-hexane:diethylether:acetic acid; 80:20:2, vol.:vol.:vol.). Radioactivity in bands corresponding to NEFA, TG, non-esterified cholesterol and CE was determined by liquid scintillation counting.

### Liver acyl-CoAs

Acyl-CoAs were extracted from liver lysate homogenates using 0.5 ml of buffer (50% 0.1 mol/l KH_2_PO_4_, 50% 2-propanol; 4°C), 30 μl saturated (NH_4_)_2_SO_4_ and 0.5 ml acetonitrile, and centrifuged (2,500 *g,* 10 min, 4°C). Acyl-CoAs were determined by liquid chromatography-mass spectrometry as described previously [16]. See ESM Methods for further details.

### Neutral TG hydrolase activity

Neutral TG hydrolase activity was measured as described previously [17], with minor modifications as described in ESM Methods.

### Mitochondria isolation and respirometry

Liver mitochondria were isolated as described previously [18] and resuspended in medium. Mitochondria were diluted in medium plus 0.2% NEFA-free BSA, 5 mmol/l glutamate, 1 mmol/l malate, or 5 mmol/l pyruvate, 5 mmol/l succinate, 1 μmol/l rotenone. Oxygen consumption rates were measured at 37°C (Oxygraph-2k; Oroboros Instruments, Innsbruck, Austria). State 3-respiration (mitochondrial respiration due to ADP supply) and state 4o-respiration (after ATP synthase inhibition by oligomycin) were induced by 2 mmol/l ADP and 1 μg/ml oligomycin. Maximal uncoupled respiration was determined after titration of carbonyl cyanide *p*-trifluoromethoxyphenylhydrazone. See ESM Methods for further details.

### RNA isolation and quantitative real-time PCR analysis

RNA isolation and quantitative real-time PCR were performed as described previously [19]. See ESM Methods for primer sequences and further details.

### VLDL secretion

Mice (8 h-fasted) were i.p. injected with 500 mg/kg BW tyloxapol in PBS. Plasma TG were determined every hour post-injection.

### Tolerance tests

Mice were i.p. injected with glucose (2 g/kg BW), insulin (0.25 U/kg BW), glucagon (140 μg/kg BW), glycerol (2 g/kg BW), pyruvate (2 g/kg BW) and glutamine (2 g/kg BW). Blood glucose levels were determined using Accu-Chek Active glucometer (Roche Diagnostics, Mannheim, Germany). See ESM Methods for further details.

### [^3^H]2-deoxy-D-glucose uptake

Mice (6 h-fasted) were i.p. injected with 30 mg glucose and 0.5 μCi glucose per 30 g BW. Radioactivity in 40 μl plasma (15, 30 and 60 min post-injection) and tissue lysates was determined by β-counting. See ESM Methods for further details.

### Quantification of metabolites by nuclear magnetic resonance spectroscopy

Liver and skeletal muscle lysates (200 μl) were mixed with methanol (400 μl), incubated at −20°C (30 min) and centrifuged. Supernatants were dried and re-dissolved in 500 μl D_2_O. ^1^H-one-dimensional-nuclear magnetic resonance experiments were performed at 310 K. Reference chemical shifts were taken from the Madison-Qingdao Metabolomics Consortium Database (http://mmcd.nmrfam.wisc.edu/) [20]. Bruker Topspin3.1 (Rheinstetten, Germany) and MestReNova10.0 software (http://mestrelab.com) were used for data acquisition, processing and analyses. See ESM Methods for further details.

### In vivo MRI for body fat

MR images of anesthetised mice were acquired by 3T-MRI (Siemens Tim-Trio, Erlangen, Germany) with an eight-channel multipurpose coil (Noras MRI products, Hoechenberg, Germany).

### Western blotting

Protein samples were separated by SDS-PAGE and transferred to polyvinylidene-difluoride membranes. Blots were incubated with primary antibodies followed by HRP-conjugated secondary antibodies. See ESM Methods for further details.

### Subcutaneous WAT sections

Tissue samples were fixed in 4% paraformaldehyde and embedded in paraffin. Tissue sections (4 μm) were stained with haematoxylin and eosin (H&E) and evaluated by light microscopy.

### Electron microscopy

Liver sections were fixed in phosphate buffer/2.5% glutaraldehyde, washed, post-fixed in phosphate buffer/OsO_4_ for 1 h and 4 × 10 min in phosphate buffer. After dehydration, tissues were infiltrated (acetone and agar 100 epoxy resin, pure agar 100 epoxy resin) for 4 h, placed in agar 100 epoxy resin (8 h), transferred into embedding moulds, and allowed to polymerise (48 h, 60°C). Sections stained with lead citrate and uranyl acetate were imaged. See ESM Methods for further details.

### Statistics

Since *Lal*^*-/-*^ mice were obviously much smaller than WT mice, in vivo experiments were not blind. In vitro experiments were blind to group assignment and outcome assessment. Statistical analyses were performed using GraphPad Prism 5.04 (GraphPad Software, San Diego, CA, USA). Significant outliers were detected by Grubb’s test (http://graphpad.com/quickcalcs/Grubbs1.cfm). Significances were determined by Student’s unpaired *t* test and Welch correction (in case of unequal variances) for two group comparison and ANOVA followed by Bonferroni correction for multiple group comparison. Data are presented as means ± SD. Differences were considered statistically significant at *p* < 0.05.

## Results

### Decreased acyl-CoA concentrations and peroxisome proliferator-activated receptor α signalling in *Lal*^*-*/-^ liver

**Fig. 1:**
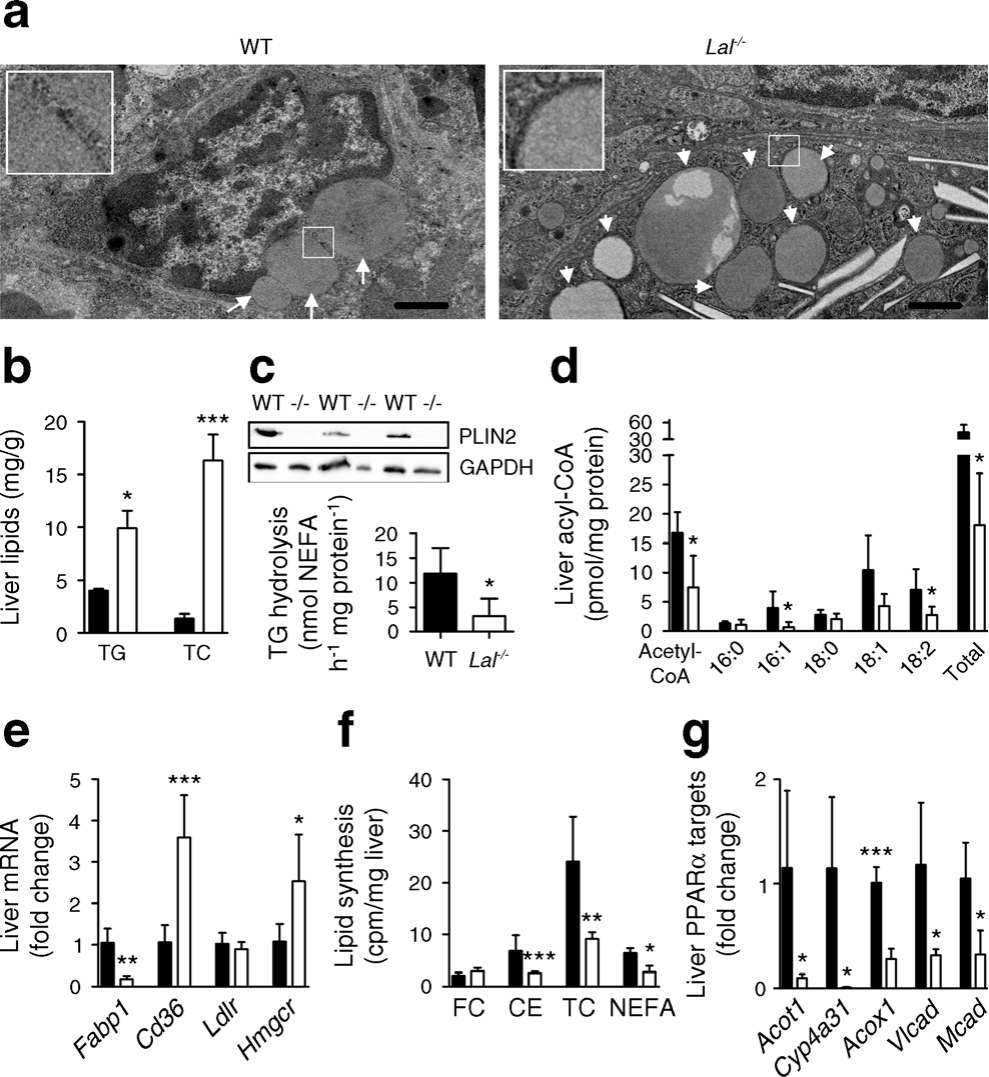
Reduced concentrations of liver acyl-CoAs in *Lal* ^*-/-*^ mice. (**a**) Electron micrographs of WT and *Lal* ^*-/-*^ liver. White arrows, lipid droplets; white arrowheads, fatty lysosomes; scale bar, 1 μm. (**b**) Hepatic TG and TC concentrations (*n* = 3). (**c**) Hepatic expression of PLIN2 with GAPDH as loading control, and hepatic neutral TG hydrolase activity. (**d**) Liver acyl-CoA concentrations (*n* = 4–5 mice aged 7–9 weeks). (**e**) Hepatic mRNA expression of fatty acid binding protein 1 (*Fabp1*), cluster of differentiation 36 (*Cd36*), LDL receptor (*Ldlr*) and 3-hydroxy-3-methylglutaryl-CoA reductase (*Hmgcr*) (*n* = 5–6). (**f**) Hepatic incorporation of i.p. injected [^14^C]acetate into lipid classes after TLC separation (*n* = 5 mice aged 17–20 weeks); FC, non-esterified cholesterol. (**g**) mRNA expression of acyl-CoA thioesterase1 (*Acot1*), cytochrome P450, family4, subfamily a, polypeptide31 (*Cyp4a31*), acyl-CoA oxidase1 (*Acox1*), very long-chain (*Vlcad*) and medium chain acyl-CoA dehydrogenase (*Mcad*; also known as *Acadm*) (*n* = 4–5). Data represent means ± SD; *p < 0.05, **p ≤ 0.01, ***p ≤ 0.001, Student’s unpaired *t* test. If not stated otherwise, mice were 12–16 weeks of age. Black bars, WT mice; white bars, *Lal* ^*-/-*^ mice

We observed severe accumulation of total cholesterol (TC) and TG in livers of *Lal*^*-*/-^ mice (Fig. 1a, b). Livers of WT mice store lipids within cytosolic lipid droplets, whereas in *Lal*^*-/-*^ mice lipids were entrapped within lysosomes distinguished by bilayer membranes (Fig. 1a). In addition, a substantial number of cholesterol crystals were visible. TG and CE could not be hydrolysed and remained entrapped within the lysosomes (Fig. 1b). Reduced protein expression of perilipin 2 (PLIN2) and reduced neutral TG hydrolase activity indicated a reduced number of cytoplasmic lipid droplets in *Lal*^*-/-*^ livers (Fig. 1c). In accordance, acyl-CoA concentrations were reduced in livers of *Lal*^*-/-*^ mice, with markedly decreased acetyl-, palmitoleoyl-, oleoyl- and linoleoyl-CoA levels (Fig. 1d). Consistently, we observed decreased mRNA expression of fatty acid binding protein 1 (*Fabp1*) (Fig. 1e). Increased mRNA expression of cluster of differentiation 36 (*Cd36*) but unaltered expression of LDL receptor (*Ldlr*) suggested increased NEFA and unchanged LDL uptake, respectively (Fig. 1e). Moreover, de novo cholesterol synthesis was induced in *Lal*^*-/-*^ livers as indicated by increased expression of hydroxymethylglutaryl-CoA reductase (*Hmgcr*) (Fig. 1e) and a moderate increase in hepatic cholesterol synthesis after i.p. injection of [^14^C]acetate (Fig. 1f). By contrast, de novo production of TG, CE and NEFA was significantly reduced in *Lal*^*-/-*^ livers (Fig. 1f). mRNA levels of selected peroxisome proliferator-activated receptor α (PPARα) target genes were markedly decreased in *Lal*^*-/-*^ compared with WT livers (Fig. 1g), which is in line with decreased acyl-CoA concentrations.

### Intact mitochondrial function despite decreased ATP concentrations in *Lal*^*-/-*^ livers

**Fig. 2:**
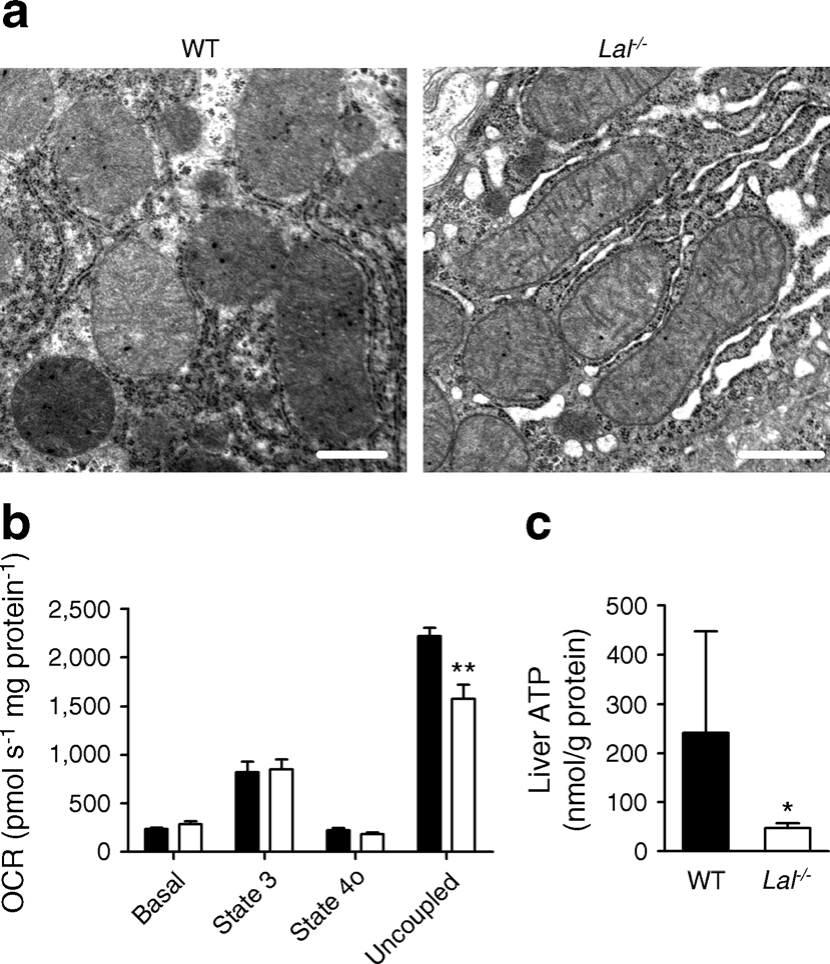
Intact mitochondria and reduced ATP concentrations in *Lal* ^*-/-*^ livers. (**a**) Electron micrographs of mitochondria in livers of WT and *Lal* ^*-/-*^ mice aged 12 weeks; scale bar, 0.5 μm. (**b**) Mitochondrial respiration of mitochondria isolated from WT and *Lal* ^*-/-*^ livers (*n* = 4–6) using 10 mmol/l succinate/1 μmol/l rotenone as substrates. Oxygen consumption rate presented as basal mitochondrial respiration in the presence of substrates (basal), increased mitochondrial respiration due to ADP supply (state 3), after ATP synthase inhibition by oligomycin (state 4o) and after carbonyl cyanide *p*-trifluoromethoxyphenylhydrazone addition (uncoupled). (**c**) Liver ATP concentrations determined by liquid chromatography-tandem mass spectrometry (*n* = 5–6 mice aged 8–12 weeks). Data represent means ± SD; *p < 0.05, **p ≤ 0.01, Student’s unpaired *t* test. Black bars, WT mice; white bars, *Lal* ^*-/-*^ mice. OCR, oxygen consumption rate

Since insufficient supply of NEFA and, consequently, acyl-CoA may affect mitochondrial function and energy production, we next investigated the hepatic mitochondria of *Lal*^*-/-*^ mice. Unaltered mitochondrial morphology (Fig. 2a) and a comparable amount of mitochondrial DNA (ESM Fig. 1a) indicated functional mitochondria. The respiratory capacity of mitochondria isolated from *Lal*^*-/-*^ livers was decreased only for the uncoupled state when succinate was provided as substrate (Fig. 2b) but unaltered with glutamate in the presence of malate (ESM Fig. 1b) or pyruvate (ESM Fig. 1c). These data suggest that the lack of substrate (acyl-CoA) might be responsible for reduced ATP concentrations in *Lal*^*-/-*^ livers (Fig. 2c).

### Diminished VLDL secretion in *Lal*^*-/-*^ mice

**Fig. 3:**
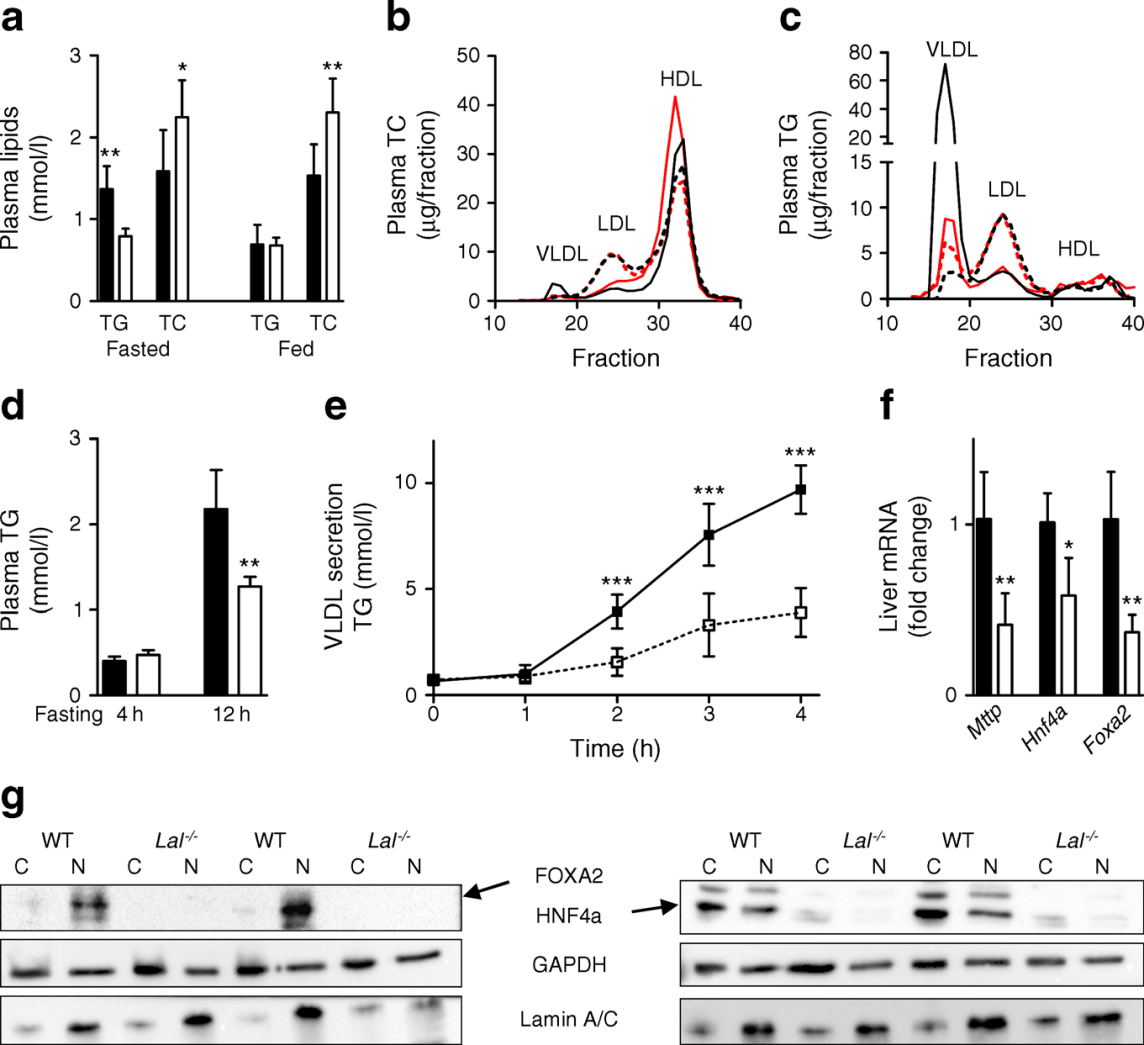
Abolished nuclear expression of HNF4α and FOXA2 and decreased VLDL synthesis in *Lal* ^*-/-*^ mice. (**a**) Plasma TG and TC concentrations in 12 h fasted (*n* = 5) and fed mice (*n* = 7–9). Distribution of (**b**) TC and (**c**) TG within plasma lipoproteins (*n* = 6); continuous lines, WT; dotted lines, *Lal* ^*-/-*^; red lines, 4 h fasting; black lines, 12 h fasting. (**d**) Plasma TG concentrations of 4 h and 12 h fasted mice (*n* = 5). (**e**) VLDL secretion of 8 h fasted mice (*n* = 4–7) after tyloxapol injection. (**f**) Hepatic mRNA expression of microsomal TG transfer protein (*Mttp*), hepatic nuclear factor 4α (*Hnf4a*) and forkhead box protein a2 (*Foxa2*) (*n* = 5–6). (**g**) Immunoblotting against liver HNF4a and FOXA2 (both in fasted state) in cytosolic (C) and nuclear (N) fractions with GAPDH and LaminA/C as loading controls. Data represent means ± SD; *p < 0.05, **p ≤ 0.01, ***p ≤ 0.001. (**a**, **d**, **f**) Student’s unpaired *t* test, (**e**) ANOVA. Mice were aged 12–16 weeks. Black bars and squares, WT mice; white bars and squares, *Lal* ^*-/-*^ mice

Since *Lal*^*-/-*^ mice suffer from progressive loss of adipose tissue, we expected lipid metabolism during fasting to be severely affected. Interestingly, fasting failed to increase circulating TG concentrations in *Lal*^*-/-*^ mice (Fig. 3a), whereas TC was increased in *Lal*^*-/-*^ mice regardless of the feeding state. Plasma lipoprotein profiles of fasted *Lal*^*-/-*^ mice revealed increased levels of LDL-cholesterol and decreased levels of HDL-cholesterol (Fig. 3b). However, in contrast to WT mice, *Lal*^*-/-*^ mice failed to induce VLDL-TG during fasting, with redistribution of TG toward the LDL fraction (Fig. 3c). Biochemical estimation confirmed the FPLC data, showing no difference after 4 h and reduced plasma TG concentrations after 12 h of fasting (Fig. 3d).

We next investigated VLDL secretion after inhibition of peripheral lipolysis by tyloxapol and found strongly reduced VLDL release in fasted *Lal*^*-/-*^ mice (Fig. 3e). As expected, *Lal*^*-/-*^ mice showed markedly increased concentrations of aminotransferases (ESM Fig. 2), an indication of liver damage. Hepatic mRNA expression of microsomal TG transfer protein (*Mttp*), a key player in VLDL assembly, was decreased (Fig. 3f). Drastically reduced mRNA and protein expression of the hepatic transcription factors hepatocyte nuclear factor 4a (*Hnf4a*) and forkhead box protein a2 (*Foxa2)* (Fig. 3f, g), which are involved in VLDL synthesis [21, 22], reflect a causal link between low expression levels of these transcription factors and decreased VLDL secretion in *Lal*^*-/-*^ livers.

### Increased insulin sensitivity in *Lal*^*-/-*^ mice

**Fig. 4:**
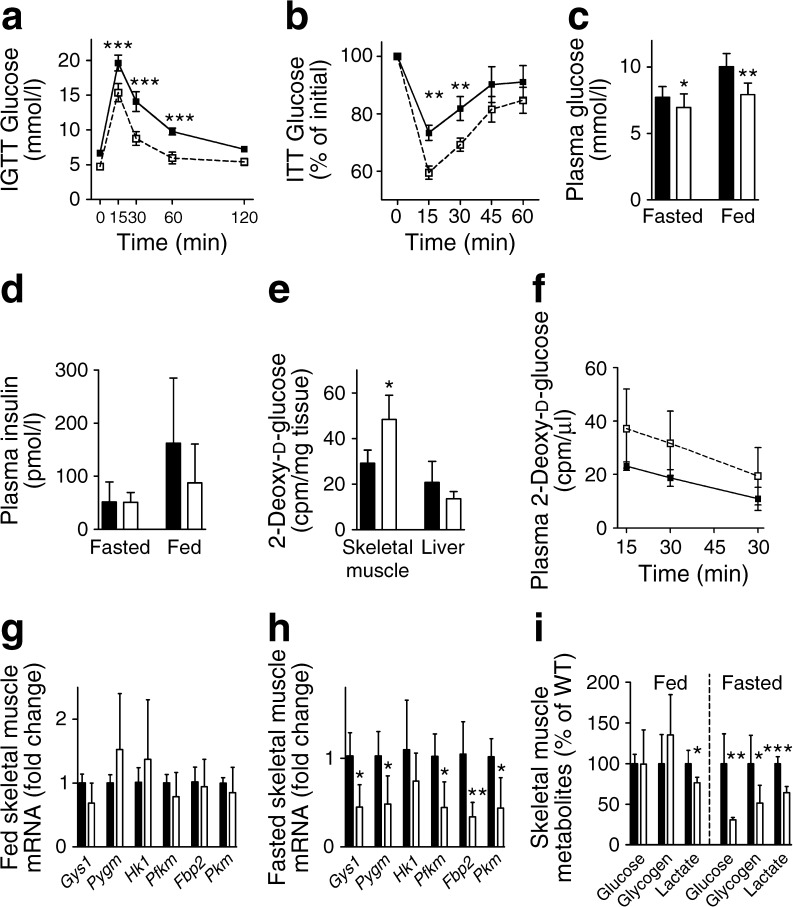
Improved glucose tolerance and insulin sensitivity in *Lal* ^*-/-*^ mice. Plasma glucose concentrations in (**a**) 6 h fasted and (**b**) 4 h fasted mice (*n* = 5) after i.p. injection of (**a**) glucose (2 g/kg) and (**b**) insulin (0.25 U/kg). Concentrations of (**c**) plasma glucose in 6 h fasted (*n* = 14) and fed (*n* = 7) mice and (**d**) insulin in 4 h fasted and fed mice (*n* = 4). (**e**) Radioactivity in skeletal muscle, liver and (**f**) plasma of mice (*n* = 5) after i.p. injection of [^3^H]2-deoxy-d-glucose. mRNA expression of glycogen synthase1 (*Gys1*), glycogen phosphorylase (*Pygm*), hexokinase 1 (*Hk1*), phosphofructokinase (*Pfkm*), fructose-biphosphatase2 (*Fbp2*) and pyruvate kinase (*Pkm*) in skeletal muscles of (**g**) fed and (**h**) 12 h fasted mice (*n* = 4–5). (**i**) Skeletal muscle metabolites of fed and 12 h fasted mice (*n* = 5). Data represent means ± SD; *p < 0.05, **p ≤ 0.01, ***p ≤ 0.001. (**a**, **b**, **f**) ANOVA, (**c**–**e**, **g**–**i**) Student’s unpaired *t* test. Mice were aged 12–16 weeks. Black bars and squares, WT mice; white bars and squares, *Lal* ^*-/-*^ mice

Severe impairment of lipid metabolism in *Lal*^*-/-*^ mice and reduced acyl-CoA and TG availability prompted us to investigate metabolic adaptations to defective lipid metabolism. Intraperitoneal glucose tolerance tests and insulin tolerance tests (ITT) revealed significantly improved glucose tolerance (Fig. 4a) and insulin sensitivity (Fig. 4b) in *Lal*^*-/-*^ mice. Plasma glucose levels were reduced (Fig. 4c), whereas insulin levels were comparable between *Lal*^*-/-*^ and WT mice (Fig. 4d). These data indicate increased usage of glucose in *Lal*^*-/-*^ mice. Accordingly, the uptake of i.p. injected [^3^H]2-deoxy-D-glucose into skeletal muscles was increased by 1.9-fold in *Lal*^*-/-*^ mice (Fig. 4e). [^3^H]2-deoxy-D-glucose uptake by *Lal*^*-/-*^ livers (Fig. 4e) as well as radioactivity in plasma (Fig. 4f) were comparable between both genotypes. mRNA expression of glycolysis genes in skeletal muscles were comparable in fed (Fig. 4g) and reduced in fasted *Lal*^*-/-*^ mice (Fig. 4h). In agreement, glucose and glycogen concentrations were decreased in fasted *Lal*^*-/-*^ mice (Fig. 4i), whereas muscle lactate was reduced in both fed and fasted states.

### Decreased plasma leptin concentrations and altered expression of hepatic adiponectin and leptin receptors in *Lal*^*-/-*^ mice

**Fig. 5:**
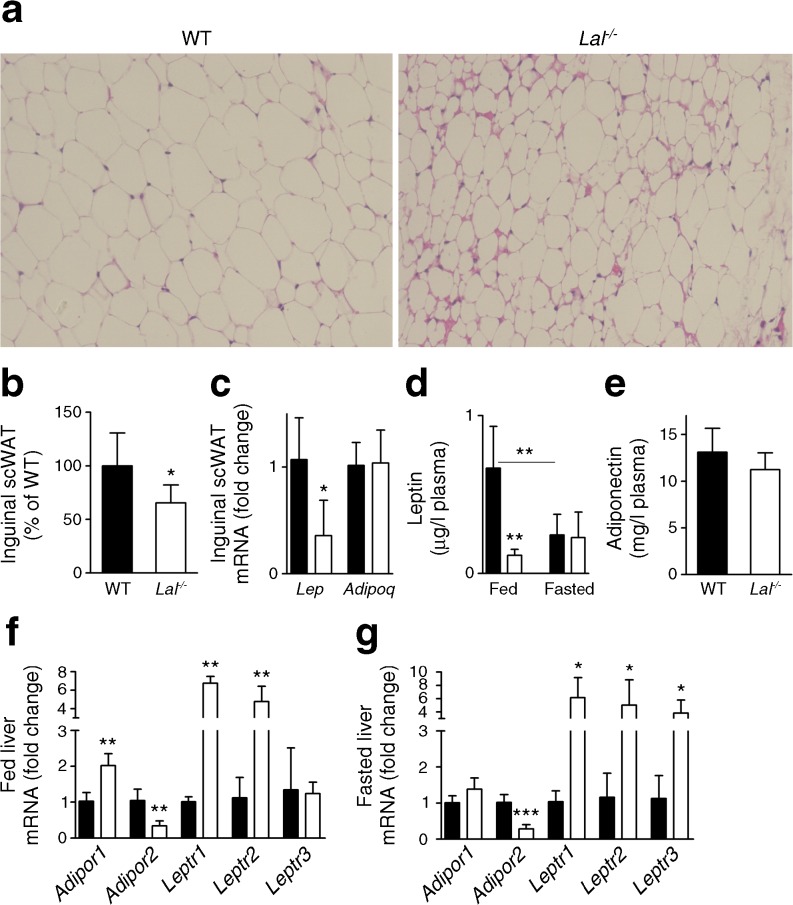
Reduced scWAT and plasma leptin concentrations in *Lal* ^*-/-*^ mice. (**a**) H&E staining of inguinal scWAT sections. (**b**) scWAT mass normalised to BW (*n* = 6). (**c**) mRNA expression of leptin (*Lep*) and adiponectin (*Adipoq*) in scWAT (*n* = 4–5). Plasma concentrations of (**d**) leptin and (**e**) adiponectin (*n* = 6). mRNA expression of the receptors of hepatic leptin (*Leptr*) and adiponectin (*Adipor*) in (**f**) fed and (**g**) 12 h fasted WT and *Lal* ^*-/-*^ mice (*n* = 3–5). Data represent means ± SD; *p < 0.05, **p ≤ 0.01; ***p ≤ 0.001, Student’s unpaired *t* test. Mice were aged 12–16 weeks. Black bars, WT mice; white bars, *Lal* ^*-/-*^ mice

Despite comparable food intake, *Lal*^*-/-*^ mice were lighter and gained less weight (ESM Fig. 3a, b). They lacked visceral fat and exhibited markedly reduced subcutaneous (sc) WAT at the age of 4 and 12 weeks (ESM Fig. 3c). H&E staining of scWAT sections revealed smaller adipocytes with reduced fat content (Fig. 5a) and we observed reduced inguinal scWAT mass (Fig. 5b) in *Lal*^*-/-*^ mice. Adipose tissue fundamentally influences systemic insulin sensitivity and glucose metabolism via the adipokines leptin and adiponectin [23]. Leptin mRNA expression was significantly decreased in scWAT of *Lal*^*-/-*^ compared with WT mice, whereas adiponectin mRNA expression was unchanged (Fig. 5c). Since fasting significantly decreases basal leptin levels [24], decreased plasma leptin concentrations (84%) in fed *Lal*^*-/-*^ mice but comparable levels as WT mice after fasting (Fig. 5d) indicate that feeding is ineffective to increase the satiety hormone leptin in *Lal*^*-/-*^ mice. Plasma adiponectin levels were comparable between both genotypes (Fig. 5e). mRNA expression of the leptin receptors *(Leptr)1* and *2* were markedly increased in livers of fed (Fig. 5f) and fasted (Fig. 5g) *Lal*^*-/-*^ mice. Expression of adiponectin receptor (*Adipor)1* mRNA was higher and expression of *Adipor2* mRNA was lower in livers of fed *Lal*^*-/-*^ mice than WT fed mice (Fig. 5f). While *Adipor1* mRNA levels were similar in fasted livers of both genotypes, transcript levels of *Adipor2* were reduced in livers of *Lal*^*-/-*^ mice compared with WT livers (Fig. 5g).

### Reduced liver glucose, glycogen and glutamine concentrations in *Lal*^*-/-*^ mice

**Fig. 6:**
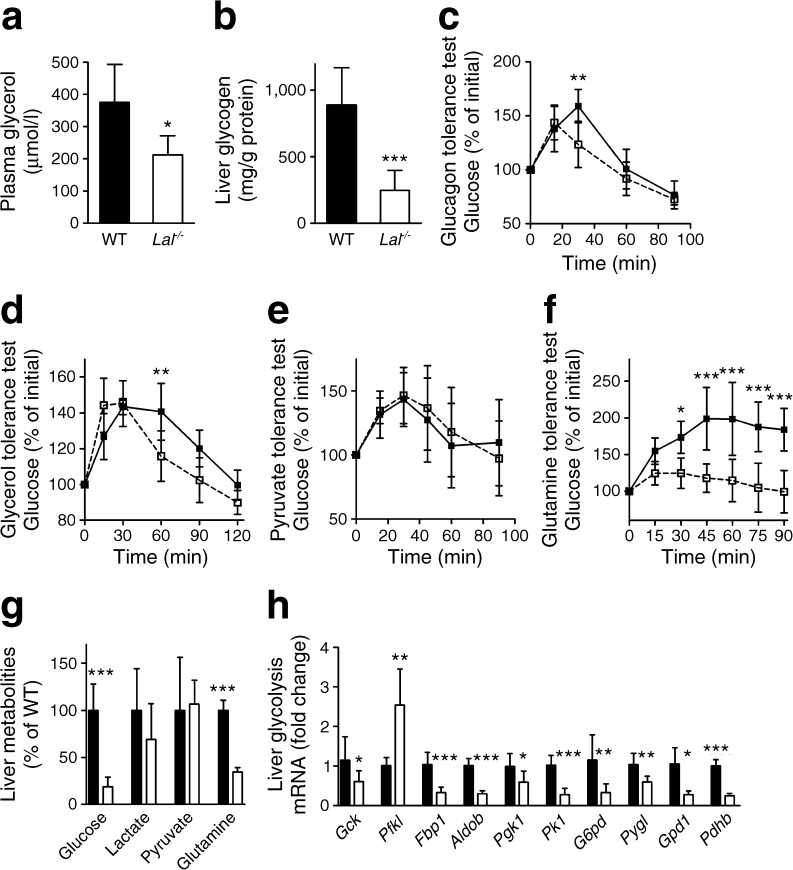
Reduced glycogen, glucose and glutamine concentrations in *Lal* ^*-/-*^ livers. (**a**) Plasma glycerol in fasted (*n* = 5) and (**b**) liver glycogen concentrations in fed mice (*n* = 8). (**c–f**) Glucose concentrations after i.p. injection of (**c**) glucagon (140 μg/kg BW), (**d**) glycerol (2 g/kg BW), (**e**) pyruvate (2 g/kg BW) and (**f**) glutamine (2 g/kg BW) in plasma of (**c**) fed and (**d**–**f**) fasted mice (*n* = 5–7). (**g**) Liver metabolites in fed mice (*n* = 6). (**h**) mRNA expression of glucokinase (*Gck*), phosphofructokinase (*Pfkl*), fructose-biphosphatase 1 (*Fbp1*), aldolase B (*Aldob*), phosphoglycerate kinase 1 (*Pgk1*), pyruvate kinase 1 *(Pk1*), glucose-6-phosphate dehydrogenase (*G6pd*; also known as *G6pdx*), glycogen phosphorylase (*Pygl*), glycerol-3-phosphate dehydrogenase1 (*Gpd1*) and pyruvate dehydrogenase (*Pdhb*) (*n* = 6–9). Data represent means ± SD; *p < 0.05, **p ≤ 0.01, ***p ≤ 0.001. (**a**, **b**, **g**, **h**) Student’s unpaired *t* test, (**c**–**f**) ANOVA. Mice were aged 12–18 weeks. Black bars and squares, WT mice; white bars and squares, *Lal* ^*-/-*^ mice

Significantly reduced plasma glycerol concentrations in fasted *Lal*^*-/-*^ mice indicate diminished peripheral lipolysis (Fig. 6a). Reduced liver glycogen concentrations in fed *Lal*^*-/-*^ mice (Fig. 6b) were confirmed by decreased mobilisation of glucose from glycogen after i.p. injection of glucagon (Fig. 6c). Reduced liver storage of glucose is a result of ineffective production or extensive usage. We therefore determined the ability of *Lal*^*-/-*^ mice to produce glucose from different carbon sources. After i.p. injection of glycerol, de novo synthesised glucose reached similar maximal values after 30 min in WT and after 15 min in *Lal*^*-/-*^ mice (Fig. 6d). Thus, glucose was cleared faster from the circulation in *Lal*^*-/-*^ mice as shown by significantly decreased levels after 60 min, implying increased systemic glucose usage. Gluconeogenesis as measured by pyruvate tolerance test was unaltered (Fig. 6e), but drastically decreased in *Lal*^*-/-*^ mice after i.p. injection of glutamine (Fig. 6f). In line, hepatic glucose and glutamine concentrations were markedly reduced in livers of fed *Lal*^*-/-*^ mice (Fig. 6g), whereas lactate and pyruvate levels were comparable to those in WT mice. Decreased hepatic glucose content may be a reason why (with the exception of liver-specific phosphofructokinase [*Pfkl*]) the mRNA expression levels of all other liver enzymes involved in glycolysis were reduced in *Lal*^*-/-*^ livers (Fig. 6h).

### PPARα activation reduces liver glucose and glycogen as well as plasma TG concentrations in *Lal*^*-/-*^ mice

**Fig. 7:**
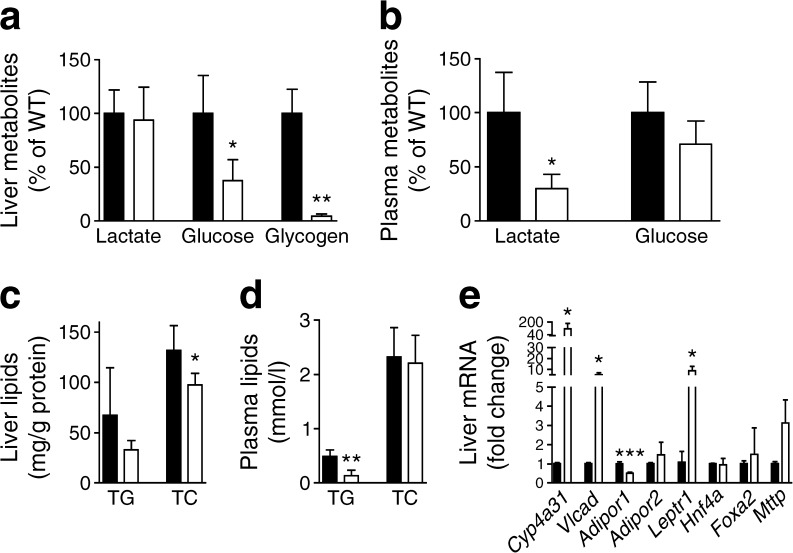
Reduced plasma TG and liver glucose and glycogen in fenofibrate-treated *Lal* ^*-/-*^ mice. Five-week-old *Lal* ^*-/-*^ mice were administered fenofibrate (0.2%) for 4 weeks. (**a**) Hepatic lactate, glucose and glycogen, and (**b**) plasma lactate and glucose concentrations (*n* = 5). TG and TC concentrations in (**c**) liver and (**d**) plasma (*n* = 4). (**e**) mRNA expression of cytochrome P450, family4, subfamily a, polypeptide 31 (*Cyp4a31*), very long-chain chain acyl-CoA dehydrogenase (*Vlcad*), adiponectin receptors (*Adipor1* and *2*), leptin receptor 1 (*Leptr1*), hepatic nuclear factor 4α (*Hnf4a*), forkhead box protein a2 (*Foxa2*) and microsomal TG transfer protein (*Mttp*) (*n* = 3–4). Data represent means ± SD; *p < 0.05, **p ≤ 0.01, ***p ≤ 0.001. Student’s unpaired *t* test. Black bars, *Lal* ^*-/-*^ mice; white bars, fenofibrate-treated *Lal* ^*-/-*^ mice

To investigate whether alterations in glucose homeostasis and VLDL synthesis in *Lal*^*-/-*^ mice are mediated by diminished PPARα activation, *Lal*^*-/-*^ mice were fed fenofibrate for 4 weeks. We observed decreased hepatic glucose and completely depleted glycogen content in fenofibrate-treated *Lal*^*-/-*^ mice (Fig. 7a). Lactate concentrations in the liver were unaffected, whereas plasma lactate levels were reduced (Fig. 7b). Moreover, fenofibrate treatment reduced liver TC and plasma TG concentrations (Fig. 7c, d). We further found induced expression of the PPARα targets *Cyp4a31* and *Vlcad* (also known as *Acadvl*), reduced *Adipor1* transcript level and increased expression of *Leptr1* (Fig. 7e). Importantly, relative mRNA expression levels of *Mttp*, *Hnf4a* and *Foxa2* remained comparable between untreated and fenofibrate-treated *Lal*^*-/-*^ mice. We conclude that defective VLDL synthesis in *Lal*^*-/-*^ mice is a consequence of diminished hepatic HNF4α/FOXA2 signalling rather than PPARα inactivation.

## Discussion

The appearance of hepatic ‘fatty lysosomes’ in insulin-sensitive *Lal*^*-/-*^ mice contrasts with hepatosteatosis, in which accumulation of cytoplasmic lipid droplets is accompanied by insulin resistance [25–27]. Liver is the main organ for VLDL assembly and secretion and thus influences whole body TG and cholesterol homeostasis [28]. Skop et al have already suggested the involvement of LAL in VLDL synthesis [29]. The authors linked the process to autophagy, since inhibition of lysosomal activity by chloroquine decreased VLDL secretion *in vitro*. Our *in vivo* data, however, indicate that defective VLDL synthesis in *Lal*^*-/-*^ mice is a consequence of decreased hepatic availability of acyl-CoAs, which then leads to downregulation of PPARα signalling, nuclear exclusion of HNFα and FOXA2, decreased *Mttp* expression, reduced TG synthesis, and eventually futile lipidation of ApoB [21, 22, 30, 31]. In general, PPARα activation mediates lipid oxidation and reduces ectopic lipid storage, thereby counteracting insulin resistance [32]. Interestingly, *Lal*^*-/-*^ mice show reduced expression of PPARα target genes, yet produce less VLDL, which contrasts with the increased secretion of VLDL observed in *Ppara*^*-/-*^ mice [33]. However, fenofibrate treatment did not normalise the phenotype but further decreased plasma TG (and hepatic glucose and glycogen) concentrations in *Lal*^*-/-*^ mice. These findings indicate that neither activation nor inactivation of PPARα per se but rather the availability of hepatic acyl-CoAs regulates VLDL synthesis and subsequent metabolic adaptations.

HNF4α, a key regulator of various metabolic pathways, is classified as an orphan receptor despite NEFA being suggested as its endogenous ligand [34]. In fact, Yuan et al demonstrated that HNF4α is selectively associated with linoleic acid in mammalian cells and in the liver of fed mice [35], indicating that linoleic acid is at least one possible endogenous ligand for HNF4α. Nuclear exclusion of HNF4α together with a 60% reduction in 18:2-CoA in livers of *Lal*^*-/-*^ mice suggest that lysosomal mobilisation of linoleic acid is involved in VLDL synthesis via the HNF4α pathway. Accordingly, protein expression of FOXA2, which is controlled by insulin signalling [36] and promotes VLDL synthesis [21], was significantly reduced in *Lal*^*-/-*^ liver. Besides reduced VLDL secretion, *Lal*^*-/-*^ mice had increased insulin sensitivity compared with their WT littermates. This finding is in accordance with studies in animal models [37, 38] and type 2 diabetes patients [39], indicating a connection between insulin resistance and VLDL overproduction. We thus propose that reduced plasma VLDL is one of the features to induce a shift from NEFA to glucose utilisation, resulting in improved insulin sensitivity as observed by enhanced uptake of glucose in skeletal muscles of these mice. Although non-significant, a 35% increase of skeletal muscle glycogen in fed *Lal*^*-/-*^ mice indicates enhanced storage of glucose for energy supply during fasting.

WAT is a primary contributor to metabolic regulation during feeding and fasting. Although lipodystrophy is generally associated with insulin resistance [40], *Lal*^*-/-*^ mice show reduced plasma glucose and enhanced glucose usage. Despite substantial loss of WAT mass in *Lal*^*-/-*^ mice, plasma concentrations of adiponectin were comparable, whereas leptin levels were profoundly decreased after feeding. These findings indicate that *Lal*^*-/-*^ mice have a constant energy demand due to the unavailability of NEFA from WAT cytosolic lipid droplets. Diminished VLDL synthesis induces depletion of liver energy storage pools as reflected by reduced liver glycogen, glucose and glutamine concentrations. Decreased liver glucose levels may be causative for lower hepatic mRNA expression of genes involved in glycolysis, particularly of *Gck* and *Pk1* (also known as *Pklr*). The liver provides glucose through gluconeogenesis from non-carbohydrate precursors during prolonged fasting [41]. *Lal*^*-/-*^ mice, however, are unable to produce glucose from external glutamine, suggesting that the degradation of glutamine to pyruvate is defective. However, gluconeogenesis per se is functional, as shown by unaltered pyruvate tolerance tests. Glycerol tolerance test revealed earlier maximal glucose levels and glucose was cleared faster from the circulation, confirming enhanced glucose utilisation in *Lal*^*-/-*^ mice.

**Fig. 8:**
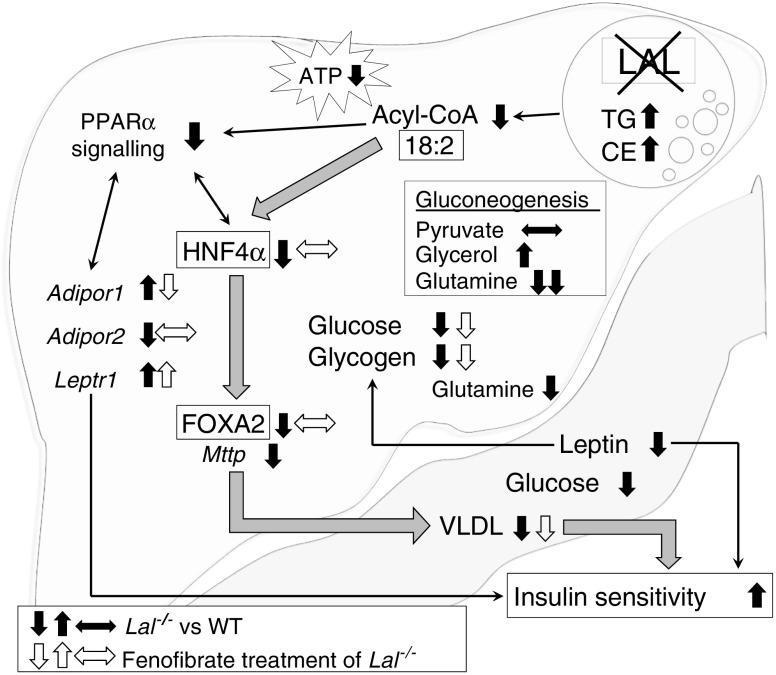
Decreased hepatic acyl-CoA availability reduces VLDL synthesis and triggers insulin sensitivity in *Lal* ^*-/-*^ mice. Inhibited hepatic lysosomal hydrolysis of CE and TG due to LAL deficiency leads to reduced abundance of cellular acyl-CoA and ATP. Consequently, decreased expression of the PPARα targets, HNF4α, FOXA2 and *Mttp* results in defective hepatic VLDL secretion. Decreased plasma glucose and leptin, and reduced liver glucose, glycogen and glutamine concentrations in fed *Lal* ^*-/-*^ mice indicate increased energy demand and extensive systemic usage of glucose. Treatment with the PPARα agonist fenofibrate further reduces plasma TG, liver glucose and glycogen concentrations in *Lal* ^*-/-*^ mice, indicating that VLDL synthesis and insulin sensitivity is PPARα-independent. Decreased VLDL production might be a consequence of reduced hepatic acyl-CoA concentrations, particularly the HNF4a ligand linoleic acid (18:2). Glucose tolerance and insulin sensitivity are increased to compensate for decreased energy availability

We conclude that defective lysosomal hydrolysis of CE and TG decreases hepatic acyl-CoA and ATP concentrations. Consequently, abolished nuclear expression of HNF4α and FOXA2 lead to reduced *Mttp* expression and attenuated VLDL secretion, which in turn induces insulin sensitivity (Fig. 8). During the past 30–40 years, our understanding of proteins and lipids that influence VLDL assembly and secretion has changed tremendously [42]. We argue that LAL should be included as another critical player, which regulates VLDL and glucose metabolism.

## Electronic supplementary material

Below is the link to the electronic supplementary material.

ESM Methods (PDF 189 kb)

ESM Fig. 1 (PDF 53 kb)

ESM Fig. 2 (PDF 58 kb)

ESM Fig. 3 (PDF 187 kb)

## Abbreviations

BW: Body weight
CE: Cholesteryl ester
CESD: Cholesteryl ester storage disease
FPLC: Fast protein liquid chromatography
H&E: Haematoxylin and eosin
ITT: Insulin tolerance test
LAL: Lysosomal acid lipase
PLIN2: Perilipin 2
PPARα: Peroxisome proliferator-activated receptor α
scWAT: subcutaneous white adipose tissue
TC: Total cholesterol
TG: Triacylglycerol
TLC: Thin-layer chromatography
WAT: White adipose tissue
WD: Wolman disease
WT: Wild-type
