# Supplementary material for: Lysosomal acid lipase regulates VLDL synthesis and insulin sensitivity in mice
Source: Diabetologia. 2016 May 6;59:1743–52. doi: 10.1007/s00125-016-3968-6 (PMC4930475; doi:10.1007/s00125-016-3968-6)
Supplement: Supplementary file 1 — (PDF 189 kb) [file 125_2016_3968_MOESM1_ESM.pdf]

Electronic Supplementary Material

Lysosomal acid lipase regulates VLDL synthesis and insulin sensitivity in mice

by B. Radovic et al.

ESM Methods

Animal experiments

Age- and sex-matched *Lal-/-* and WT littermates (Hong Du, Indiana University School of Medicine, Indianapolis, IN) were between 12 and 16 weeks of age when experiments were performed. Age of mice used for specific experiments are indicated in the figure legends. Mice were back-crossed to C57BL/6J background. Mice were maintained in a clean environment with unlimited access to chow diet and water in a regular light-dark cycle (12 h light/12 h dark). Food intake was determined in individually housed mice. Body weights were determined in 8, 15, and 19 weeks old mice. For fenofibrate treatment, 5-weeks-old *Lal-/-* mice were administered 0.2% fenofibrate mixed with chow diet for 4 weeks *ad libitum*. All animal experiments were approved by the Austrian Federal Ministry of Science, Research, and Economy, Vienna, Austria (BMWF-66.010/0057-II/3b/2011; BMWF-66.010/0159-II/3b/2012; BMWF-66.010/0085-II/3b/2013).

Plasma parameters

Approximately 30 mg of liver were homogenized in lysis buffer. Lysates containing 500 µg protein were normalized with the appropriate amount of lysis buffer to the same volume and lipids were isolated by chloroform:methanol (2:1, v:v) extraction (Folch). Thereafter, extracts were centrifuged at 2,600 x *g* for 15 min at 4°C, the organic phase was mingled with 200 µl 2 % Triton X-100 in chloroform, vortexed, and dried under the stream of nitrogen gas. Thereafter, the samples were redissolved in 100 µl ddH$_2$O. Mice were fasted for 12 h before 100 µl blood were drawn from *v. facialis*. Plasma was separated by centrifugation at 5,200 x *g* for 7 min at 4°C. TG, TC, FC, and glycerol concentrations were measured in plasma and

extracted liver lysates using enzymatic test kits (Triglycerides FS, Cholesterol FS, Free Cholesterol FS; DiaSys, Holzheim, Germany; Free Glycerol: Sigma-Aldrich, St. Louis, MO) according to manufacturers' instructions. Markers of liver injury alanine aminotransferase (ALT) (Roche Diagnostics, Mannheim, Germany) and aspartate aminotransferase (AST) (Thermo Electron Corporation, Louisville, CO) were measured enzymatically.

Fast protein liquid chromatography (FPLC)

Plasma from 5 mice per genotype were pooled and diluted with PBS (1:4). Two hundred µl of each plasma pool was injected onto a Superose 6 column (Amersham Biosciences, Piscataway, NJ) and separated by FPLC (Pfizer Pharma, Karlsruhe, Germany). The lipoproteins were eluted with 10 mM Tris-HCl, 1 mM EDTA, 0.9% NaCl, and 0.02% NaN3 (pH 7.4). Forty eight fractions of 0.5 ml each were collected, and TG and TC concentrations were assayed enzymatically. To enhance sensitivity, reaction buffers were supplemented with sodium 3,5-dichloro-2-hydroxy-benzenesulfonate.

Quantification of plasma insulin, leptin, and adiponectin

Blood of fed and 6 h-fasted mice, respectively, were drawn from *v. facialis* and plasma was isolated for determination of hormones by ELISA kits (leptin and adiponectin: Merck Millipore, Darmstadt, Germany; insulin: Mercodia, Uppsala, Sweden), according to manufacturer's protocol.

ATP determination by LC-MS/MS analysis

Polar metabolites from mouse liver were extracted as described [1]. HPLC was performed on a 1100 Agilent capillary LC (Agilent Technologies, Santa Clara, California, USA) equipped with a polyhydroxyethyl (HILIC) column (PolyLC Inc, Columbia, MD). Solvent A (10 mM ammonium acetate in water) and solvent B (10 mM ammonium acetate in 90% acetonitrile) were used as follows: 0 - 12 min isocratic 100% B, 12 - 17 min gradient (100% B to 0% B and 0% A to 100% A), 17 - 30 min isocratic 100 A, 30 - 35 min gradient (100% A to 0% A

and 0% B to 100% B), and 35 - 45 min isocratic 100% B. Injection volume was 1 µl with a flow of 10 µl per min. Selected ions and their fragments were detected using TSQ Quantum Ultra Mass Spectrometer (Thermo Fisher Scientific, Waltham, MA) in negative mode with voltage of -2500 V. Vaporizer temperature was set to 38°C, sheath gas to 6, capillary temperature to 200°C, aux gas pressure to 1, tube lens offset to -158, and skimmer offset to 12. Metabolites were monitored by their negative selected reaction monitoring pairs.

Determination of liver acyl-CoAs

Acyl-CoAs were determined by on-line solid phase extraction liquid chromatography-mass spectrometry as described previously [2]. Acyl-CoAs (internal standards of 0.5 nmol/ml C17:0 CoA, 0.6 nmol/ml $^{13}C_2$-acetyl-CoA, and 0.6 nmol/ml $^{13}C_3$-malonyl-CoA) were extracted from liver lysates using 0.5 ml of prechilled (4°C) buffer (50% 0.1 m KH_2PO4 and 50% 2-propanol). Lysates were homogenized on ice for 10–20 s using an ultrasonic homogenizer. Subsequently, 30 µl of saturated aqueous $(NH_4)_2SO_4$ and 0.5 ml of acetonitrile were added. The homogenate was vigorously mixed and centrifuged at 2,500 x *g* for 10 min at 4°C, and the supernatant was transferred to autosampler vials. Extracts were stored at −80°C. Analyses were performed on an Ultimate 3000 System (Dionex, LC Packings, Sunnyvale, CA) consisting of an autosampler with cooled tray and a column oven with a switching unit coupled to an LTQ Orbitrap XL (Thermo Scientific, Waltham, MA). A Phenomenex Strata X 2.0 × 20-mm cartridge (Torrance, CA) and a Waters XBridge column (2.1 × 50 mm, 2.5 µm) (Milford, MA) were used for on-line solid phase extraction and as analytical column, respectively. Positive electrospray ionization-mass spectrometry was performed by high resolution mass spectrometry (scan range 150–2000 m/z, resolution 60,000). Compound identities were confirmed using accurate mass, tandem mass spectrometry, and retention time.

Neutral TG hydrolase activity

Neutral TG hydrolase activity was measured as described [3] with minor modifications. Liver tissues were lysed with 100 µl of lysis buffer (100 mM potassium phosphate, 250 mM sucrose, 1 mM EDTA, 0.1 mM DTT, pH 7), sonicated on ice twice for 10 s with 1 min interval, and centrifuged at 1,000 x g and 4°C for 10 min. The supernatant was again centrifuged at 20,000 x g for 30 min at 4°C. Protein concentrations were measured using a Lowry assay (BioRad Laboratories, Hercules, CA). The TG substrate contained 300 nmol triolein/sample, 0.5 µCi/sample [9,10-^{3}H(N)]-triolein (Perkin Elmer, Waltham, MA), 15 µg/sample phosphatidylcholine/phosphatidylinositol (3:1), and NEFA-free BSA at a final concentration of 2%. Fifty µg of protein from liver lysates were mixed with 100 µl of substrate and incubated in a water bath for 1 h at 37°C. The reaction was stopped by the addition of 3.25 ml stop solution (methanol:chloroform:n-heptane, 10:9:7, v:v:v) and 1 ml of 0.1 M potassium carbonate, 0.1 M boric acid (pH 10.5). The tubes were vortexed for 10-15 s and centrifuged at 1700 x *g* for 15 min at 4°C. The radioactivity in 1 ml of the upper phase was determined by liquid scintillation counting, and the release of FAs was calculated.

Mitochondria isolation and respirometry

Mouse liver mitochondria were isolated using a Potter Elvehjem homogenizer as previously described [4] and resuspended in respiration medium containing 220 mM mannitol, 70 mM sucrose, 10 mM KH_2PO_4, 5 mM $MgCl_2$, 2 mM HEPES and 1.0 mM EGTA, pH 7.2. For normalization, total mitochondrial protein concentrations were quantified. Freshly isolated mitochondria (0.3 mg/chamber) were diluted in respiration medium supplemented with 0.2% FA-free BSA and either 5 mM glutamate, 1 mM malate, and 5 mM pyruvate to assess complex I or 5 mM succinate and 1 µM rotenone to assess complex II of the respiratory chain, respectively. Oxygen consumption rates were measured at 37°C using the Oxygraph-2k polarographic oxygen sensor (Oroboros® Instruments, Innsbruck, Austria). Basal oxygen consumption rates were recorded in the presence of substrates after closing the chamber. State 3-respiration was induced by addition of 2 mM ADP. State 4o-respiration was measured following the addition of 1 µg/ml oligomycin. Maximal uncoupled respiration was

observed after titration of the uncoupling agent carbonyl cyanide *p*-trifluoromethoxyphenylhydrazone.

RNA isolation and quantitative real-time PCR analysis

Total RNA was isolated using TriFast reagent according to the manufacturer's protocol (Peqlab, Erlangen, Germany). One µg of total RNA was reverse transcribed using the High Capacity cDNA Reverse Transcription Kit (Applied Biosystems, Carlsbad, CA). Quantitative real-time PCR was performed on a Roche LightCycler 480 (Roche Diagnostics, Palo Alto, CA) using the QuantifastTM SYBR® Green PCR kit (Qiagen, Hilden, Germany). Samples were analyzed in duplicate and normalized to the expression of cyclophilin A as reference gene. Expression profiles and associated statistical parameters were determined using the $2^{-\Delta\Delta CT}$ method. Primer sequences were used as follows:

Acot1: Fw: GGCTGGGAATGGAGTTTCAT, Rev: GCTATCCAAGAAAAGTGCCAGG,

Acox1: Fw: TCCAGACTTCCAACATGAGGA, Rev: CTGGGCGTAGGTGCCAATTA;

Adipoq: Fw: GGAGATGCAGGTCTTCTTGG, Rev: CGAATGGGTACATTGGGAAC;

Adipor1: Fw: AATGGGGCTCCTTCTGGTAAC, Rev: GGATGACTCTCCAACGTCCCT;

Adipor2: Fw: GCCAAACACCGATTGGGGT, Rev: GGCTCCAAATCTCCTTGGTAGTT;

Aldob: Fw: GAAACCGCCTCCAAAGGATAA, Rev: GAGGGTCTCGTGGAAAACCAT;

Cd36: Fw: GCAGGTCTATCTACGCTGTG, Rev: GGTTGTCTGGATTCTGGAGG;

CycloA: Fw: CCATCCAGCCATTCAGTCTT, Rev: TTCCAGGATTCATGTGCCAG;

Cyp4a: Fw: GGTGACCAAGAACTACAGGA, Rev: GCCTGTCCATTCAACAAGAG;

Fabp1: Fw: ATGAACTTCTCCGGCAAGTACC, Rev: GGTCCTCGGGCAGACCTAT;

Fbp1: Fw: CACCGCGATCAAAGCCATCT, Rev: CCAGTCACATTGGTTGAGCCA;

Fbp2: Fw: ACCCGTTACGTTATGGAAAAGG, Rev: GGCAGTCAGCATCGAGTTGAG;

Foxa2: Fw: TCCGACTGGAGCAGCTACTAC, Rev: GCGCCCACATAGGATGACA;

G6pc: Fw: CGACTCGCTATCTCCAAGTGA, Rev: GGGCGTTGTCCAAACAGAAT;

Gck: Fw: TGAGCCGGATGCAGAAGGA, Rev: GCAACATCTTTACACTGGCCT;

Gpd1: Fw: GCCTTCGCCAAGCTCTTCTG, Rev: TAGCAGGTCGTGATGAGGTCTG;

Gys1: Fw: GAACGCAGTGCTTTTCGAGG, Rev: CCAGATAGTAGTTGTCACCCCAT;

Hk1: Fw: TGATCGCCTGCTTATTCACGG, Rev: AACCGCCTAGAAATCTCCAGA;

Hnf4a: Fw: GTGCGAACCTCAATTCATCC, Rev: TGTCTACCACACATTGTCGG;

Hmgcr: Fw: CTATTGCACCGACAAGAAGCCT, Rev: GCCATCACAGTGCCACATACAA;

Ldlr: Fw: CATGTCTGTCACCTGTCAGTCC, Rev: CTTGTCCAAGCTGATGCACTCC;

Lep: Fw: TGAGTTTGTCCAAGATGGACC, Rev: GCCATCCAGGCTCTCTGG;

Leptr1: Fw: AATGACGCAGGGCTGTATGT, Rev: ATGGACTGTTGGGAAGTTGG;

Leptr2: Fw: AATGACGCAGGGCTGTATGT, Rev: TCAGGCTCCAGAAGAAGAGG;

Leptr3: Fw: TGAAGATGATGGAATGAAGTGG, Rev: AGTTGGCACCTTATTTTGAGGT;

Mcad: Fw: GCAACTGCCCGCAAGTTT, Rev: TACTCCCCGCTTTTGTCATATTC;

Mttp: Fw: GTCAACAGAGAGGCGAGAAG, Rev: CTAGCCAAGCCTCTCTTGAG;

Pdhb: Fw: CGGTGCAGTTGACAGTTCGT, Rev: TCTTCCCCAAGCAGAAAAACTTT;

Pfkl: Fw: GGAGGCGAGAACATCAAGCC, Rev: GCACTGCCAATAATGGTGCC;

Pfkm: Fw: CATCGCCGTGTTGACCTCT, Rev: CCCGTGAAGATACCAACTCGG;

Pgk: Fw: CTGTGGTACTGAGAGCAGCAAGA, Rev: CAGGACCATTCCAAACAATCTG;

Pk1: Fw: CTTGCTCTACCGTGAGCCTC, Rev: ACCACAATCACCAGATCACC;

Pkm: Fw: GGCTGAATTTCTCTCATGGAACC, Rev: CACCGCAACAGGACGGTAG;

Pygl: Fw: GAGAAGCGACGGCAGATCAG, Rev: CTTGACCAGAGTGAAGTGCAG;

Pygm: Fw: CAAATCAGCGTTCGTGGCTTA, Rev: CCACATTGCGATCCTTGACCA;

Vlcad: Fw: CTACTGTGCTTCAGGGACAAC, Rev: CAAAGGACTTCGATTCTGCCC;

Glucose tolerance test

Six hour-fasted mice were administered (orally and i.p.) with 2 g/kg BW of glucose in PBS. Thereafter, blood glucose levels were measured at 0, 15, 30, 60, and 120 min using Accu-Chek® Active glucometer and glucose strips (Roche Diagnostics GmbH, Mannheim, Germany).

Insulin tolerance test

Four hour-fasted mice were i.p. injected with 0.25 IU/kg BW of insulin in PBS (100 IU/ml stock; Actrapid Novo Nordisk, Wien, Austria). Blood glucose levels were measured 0, 15, 30, 45, and 60 min post-injection using Accu-Chek® Active glucometer and glucose strips (Roche Diagnostics GmbH).

Glucagon tolerance test

Mice in fed state were injected i.p. with 140 µg/kg BW of glucagon (GlucaGen® HypoKit 1 mg, Novo Nordisk) in PBS. Blood glucose levels were measured 0, 15, 30, 60, and 90 min post-injection using Accu-Chek® Active glucometer and glucose strips (Roche Diagnostics GmbH).

Glycerol, pyruvate, and glutamine tolerance tests

Sixteen hours-fasted mice were injected i.p. with 2 g/kg BW of glycerol, pyruvate, and glutamine in PBS, respectively. Blood glucose levels were measured at indicated times using Accu-Chek® Active glucometer and glucose strips (Roche Diagnostics GmbH).

Quantification of metabolites by NMR spectroscopy

Phosphate-buffered solution was prepared by dissolving 5.56 g anhydrous NaH_2PO_4, 0.4 g 3(trimethylsilyl)propionic acid-2,2,3,3-d_4 sodium salt (TSP), and 0.2 g NaN_3 in 400 ml deionized water and adjusted to pH 7.4 with 1 M NaOH and HCl. Upon addition of deionized water to a final volume of 500 ml, the pH was readjusted to pH 7.4 with 1 M NaOH and HCl. The mixture of 200 µl liver or skeletal muscle lysate and 400 µl methanol was incubated at -20°C for 30 min and centrifuged at 18,000 x *g* for 30 min to pellet proteins. Supernatants were mixed with 500 µl phosphate buffer, samples were dried, dissolved in 500 µl D_2O, and transferred to 5 mm NMR tubes. NMR experiments were performed at 310 K on a Bruker Avance III 500MHz spectrometer equipped with a TXI probe head. The one-dimensional (1D) CPMG (Carr−Purcell−Meiboom−Gill) pulse sequence (cpmgpr1d, 73728 points in F1, 12019.230 Hz spectral width, 2048 transients, recycle delay 4 s), with water suppression

using presaturation, was used for ^{1}H 1D NMR experiments. Metabolite reference chemical shifts were taken from the Madison-Qingdao Metabolomics Consortium Database (http://mmcd.nmrfam.wisc.edu/) [5] and all metabolites were cross-checked using reference compounds. Bruker Topspin version 3.1 (Rheinstetten, Germany) and MestReNova version 10.0 (http://mestrelab.com) software packages were used for NMR data acquisition, processing, and analyses. Metabolite concentrations were determined using TSP as internal standard.

In vivo MR imaging for body fat

MR images of anesthetized mice were acquired by 3T MRI (Siemens Tim-Trio, Erlangen, Germany) with an eight-channel multipurpose coil (Noras MRI products, Hoechenberg, Germany) to maximize signal-to-noise ratio.

Western blotting

Protein samples (40 µg protein/lane) of whole liver lysates or nuclear and cytoplasmic fractions, respectively, fractionated by Subcellular Protein Fractionation Kit (Thermo Fisher Scientific, Waltham, MA), were separated by 15% SDS-polyacrylamide gel electrophoresis and transferred to polyvinylidene difluoride membranes. Blots were incubated with anti-rabbit polyclonal antibodies against HNF4α and Lamin A/C (1:1,000; Santa Cruz Biotechnology, Inc, Santa Cruz, CA), FOXA2 and GAPDH (1:1,000; Cell Signaling Technology, Danvers, MA), and an anti-guinea pig polyclonal antibody against PLIN2 (GP40; 1:5,000; Progen, Heidelberg, Germany). Horseradish peroxidase-conjugated goat anti-rabbit (1:2,000; Dako, Glostrup, Denmark) and anti-guinea pig (1:10,000; Southern Biotech, Birmingham, AL,) antibodies were visualized by enhanced chemiluminescence detection (ECL Plus; Thermo Scientific, Rockford, IL or Clarity™ Western ECL Substrate; Bio-Rad, Vienna, Austria) using the BioRad ChemiDoc™ MP Imaging System (BioRad Laboratories Inc, Hercules, CA).

ScWAT sections

Tissue samples were fixed in 4% paraformaldehyde and embedded in paraffin. Tissue sections (4μm) were stained with H&E and evaluated by light microscopy.

Electron microscopy

Liver tissues were fixed in 0.06 M phosphate buffer (pH 7.2) containing 2.5% glutardialdehyde for 90 min at room temperature, washed two times in 0.06 M phosphate buffer for 10 min and post-fixed in 1% osmium tetroxide in 0.06 M phosphate buffer (pH 7.2) for 1 h. Thereafter, the tissues were washed four times in 0.06 M phosphate buffer (pH 7.2) for 10 min each. After dehydration for 20 min in 50, 70, 90, and 100% cold acetone, the tissues were infiltrated with 100% acetone and agar, 100 epoxy resin, and pure agar 100 epoxy resin (2:1, 1:1, 1:2) mixtures for 4 h, respectively. Thereafter, they were placed in agar 100 epoxy resin for 8 h at room temperature, transferred into embedding molds, and allowed to polymerize at 60°C for 48 h. Ultrathin sections (75 nm) were cut by a Leica Ultramicrotome (Leica Microsystems Inc., Mannheim, Germany) and stained with lead citrate for 5 min and with uranyl acetate for 15 min. Images were taken using a Philips CM 10 or a FEI Tecnai G2 20 transmission electron microscope (FEI, Eindhoven, Netherlands) equipped with a Gatan ultrascan 1000 CCD camera. Acceleration voltage was 120 kV.

References

[1] Yuan M, Breitkopf SB, Yang X, Asara JM (2012) A positive/negative ion-switching, targeted mass spectrometry-based metabolomics platform for bodily fluids, cells, and fresh and fixed tissue. Nat Protoc 7:872-881

[2] Magnes C, Suppan M, Pieber TR, et al. (2008) Validated comprehensive analytical method for quantification of coenzyme A activated compounds in biological tissues by online solid-phase extraction LC/MS/MS. Anal Chem 80:5736-5742

[3] Schweiger M, Eichmann TO, Taschler U, Zimmermann R, Zechner R, Lass A (2014) Measurement of lipolysis. Methods Enzymol 538:171-193

[4] Frezza C, Cipolat S, Scorrano L (2007) Organelle isolation: functional mitochondria from mouse liver, muscle and cultured fibroblasts. Nat Protoc 2:287-295

[5] Cui Q, Lewis IA, Hegeman AD, et al. (2008) Metabolite identification via the Madison Metabolomics Consortium Database. Nat Biotechnol 26:162-164
