# Supplementary material for: Lysosomal acid lipase regulates VLDL synthesis and insulin sensitivity in mice
Source: Diabetologia. 2016 May 6;59:1743–52. doi: 10.1007/s00125-016-3968-6 (PMC4930475; doi:10.1007/s00125-016-3968-6)
Supplement: Supplementary file 2 — (PDF 53 kb) [file 125_2016_3968_MOESM2_ESM.pdf]

Electronic Supplementary Material

Lysosomal acid lipase regulates VLDL synthesis and insulin sensitivity in mice

by B. Radovic et al.

ESM Figure 1 (relates to Figure 2)

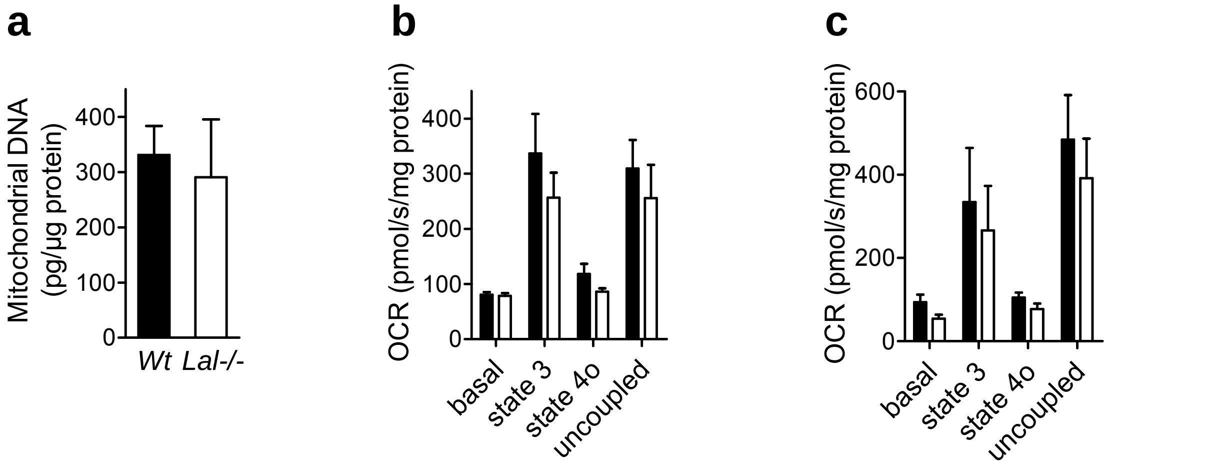

ESM Fig. 1: Comparable mitochondrial number and *ex vivo* oxygen consumption in *Lal-/-* livers. (**a**) DNA concentrations of liver mitochondria isolated from 8-10 weeks old *Wt* (black bar) and *Lal-/-* mice (white bar) (n=6). Oxygen consumption rates of extracted mitochondria using (**b**) glutamate/malate and (c) glutamate/pyruvate as substrates, respectively. Data represent mean values (n=4) + SD.
