# Supplementary material for: Lysosomal acid lipase regulates VLDL synthesis and insulin sensitivity in mice
Source: Diabetologia. 2016 May 6;59:1743–52. doi: 10.1007/s00125-016-3968-6 (PMC4930475; doi:10.1007/s00125-016-3968-6)
Supplement: Supplementary file 3 — (PDF 58 kb) [file 125_2016_3968_MOESM3_ESM.pdf]

Electronic Supplementary Material

Lysosomal acid lipase regulates VLDL synthesis and insulin sensitivity in mice

by B. Radovic et al.

ESM Figure 2 (relates to Figure 3)

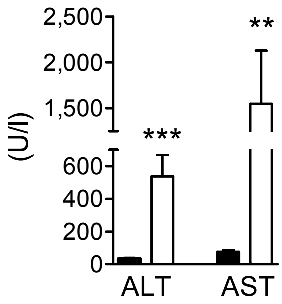

ESM Fig. 2: Increased alanine aminotransferase (ALT) and aspartate aminotransferase (AST) concentrations in *Lal-/-* mice. Plasma values of ALT and AST in 10-14 weeks old *Wt* (black bar) and *Lal-/-* (white bar) mice. Data represent mean values (n=5) + SD. $^{**}p \leq 0.01$, $^{***}p \leq 0.001$.
