# Supplementary material for: Lysosomal acid lipase regulates VLDL synthesis and insulin sensitivity in mice
Source: Diabetologia. 2016 May 6;59:1743–52. doi: 10.1007/s00125-016-3968-6 (PMC4930475; doi:10.1007/s00125-016-3968-6)
Supplement: Supplementary file 4 — (PDF 187 kb) [file 125_2016_3968_MOESM4_ESM.pdf]

Electronic Supplementary Material

Lysosomal acid lipase regulates VLDL synthesis and insulin sensitivity in mice

by B. Radovic et al.

ESM Figure 3 (relates to Figure 5)

a

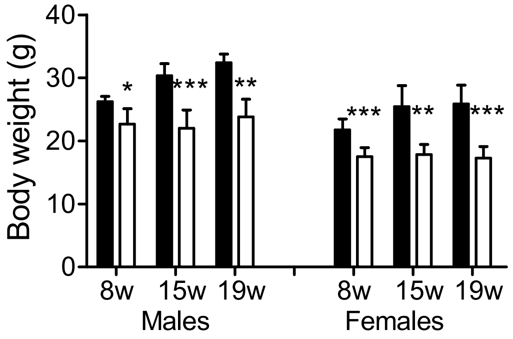

b

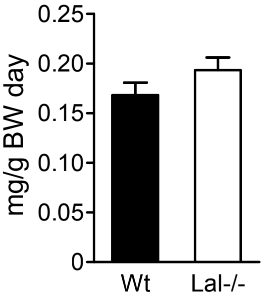

c

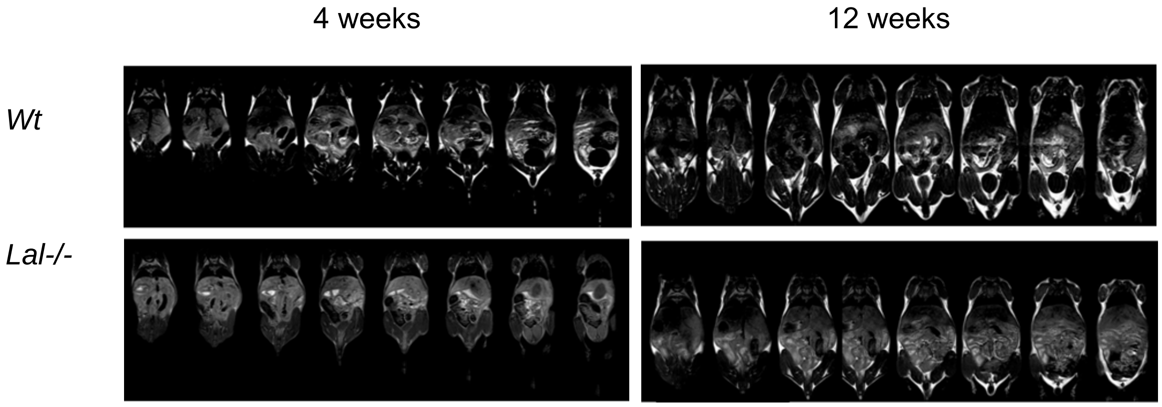

ESM Fig. 3: Decreased body weight and fat mass in *Lal-/-* mice. (**a**) Body weights of 8, 15, and 19 weeks old Wt (black bars; n=3-8) and *Lal-/-* (white bars; n=4-8) mice. (**b**) Food intake normalized to body weight of 8 weeks old *Wt* (black bar; n=6) and *Lal-/-* (white bar, n=6) mice. Animals were housed individually and had ad libitum access to standard chow diet and water. Data represent mean values + SD. *$p<0.05$, **$p\leq0.01$, ***$p\leq0.001$. (**c**) MRI cross-sectional images of 4 week old *Wt* and *Lal-/-* littermate; the same mice were scanned again at the age of 12 weeks. Bright areas represent adipose tissues.
